# The electric properties of extracellular media affect cable properties of neurons

**DOI:** 10.1186/1471-2202-14-S1-P106

**Published:** 2013-07-08

**Authors:** Claude Bedard, Alain Destexhe

**Affiliations:** 1 UNIC, CNRS, Gif-sur-Yvette, France

## 

Cable theory has been developed over the last decades, usually assuming that the extracellular space around membranes is a perfect resistor. However, extracellular media may display more complex electrical properties due to various phenomena, such as polarization, ionic diffusion or capacitive effects, but their impact on cable properties is not known. Here, we generalize cable theory for membranes embedded in arbitrarily complex extracellular media. We outline the generalized cable equations, then consider specific cases. The simplest case is a resistive medium, in which case the equations recover the traditional cable equations. We show that for more complex media, for example in the presence of ionic diffusion, or polarization, the impact on cable properties such as voltage attenuation can be significant. We illustrate this numerically always by comparing the generalized cable to the traditional cable. We conclude that the nature of intracellular and extracellular media may have a strong influence on cable filtering as well as on the passive integrative properties of neurons.

